# Physical robustness and resilience among long-lived female siblings: a comparison with sporadic long-livers

**DOI:** 10.18632/aging.103618

**Published:** 2020-07-11

**Authors:** Angéline Galvin, Svetlana Ukraintseva, Konstantin Arbeev, Mary Feitosa, Kaare Christensen

**Affiliations:** 1 Epidemiology, Biostatistics, and Biodemography, Department of Public Health, University of Southern Denmark, Odense, Denmark; 2 Center for Population Health and Aging, Duke University, Durham, NC 27708, USA; 3 Division of Statistical Genomics, Department of Genetics, Washington University School of Medicine, St. Louis, MO 63110, USA; 4 The Danish Aging Research Center, Department of Public Health, University of Southern Denmark, Odense, Denmark

**Keywords:** aging, family longevity, robustness, resilience, survival

## Abstract

Long-lived individuals are central in studies of healthy longevity. However, few pro-longevity factors have been identified, presumably because of “phenocopies”, i.e. individuals that live long by chance. Familial longevity cases may include less phenocopies than sporadic cases and provide better insights into longevity mechanisms. Here we examined whether long-lived female siblings have a better ability to avoid diseases at ages 65+ (proxy for “robustness”) and/or survive to extreme ages (proxy for “resilience”) compared to sporadic long-livers. A total of 1,156 long-lived female siblings were selected from three nationwide Danish studies and age-matched with sporadic long-lived female controls. Outcomes included cumulative incidence of common health disorders from age 65 and overall survival. Long-lived female siblings had lower risks of some but not all health conditions, most significantly, depression (OR=0.74; 95%CI=0.62-0.88), and less significantly hypertensive (OR=0.84; 95%CI=0.71-0.99) and cerebrovascular (OR=0.73; 95%CI=0.55-0.96) diseases. They also had consistently better survival to extreme ages (HR=0.71; 95%CI= 0.63-0.81) compared to sporadic long-livers. After adjustment for the diseases, the association with mortality changed only marginally suggesting central role of better physiological resilience in familial longevity. Due to their consistently better resilience, familial longevity cases could be more informative than sporadic cases for studying mechanisms of healthy longevity.

## INTRODUCTION

Long-lived individuals are central in studies of the determinants of a long and healthy life. Both genetic and environmental factors contribute to longevity, and research is increasingly more focused on the genetic part [1–4]. As highlighted in several Scandinavian twin studies, longevity clusters in families, which suggests the existence of genetic variants for survival [5–7]. These studies showed that lifespan is partly heritable, and that the heritability increases with age. One study indicated a genetic influence on human longevity, underscoring minimal genetic effects on lifespan for people under age 60 and then moderate genetic effects for people aged 60 and over [6]. However, few genetic or other pro-longevity factors have been identified, possibly due to “phenocopies” - those individuals that live long by chance. Some studies reported better health in long-lived siblings compared to sporadic long-livers, including a lower prevalence of Alzheimer’s disease and related disorders, diabetes, depression, heart failure and osteoporosis [8, 9]. One Dutch longitudinal study found better survival in long-lived siblings compared to sporadic long-livers [10]. However, these studies did not evaluate disease risk and survival in the same population and, therefore, were not able to examine the interplay between indicators of robustness and resilience, which is the focus of this study.

Physical robustness could be defined as the ability to resist a deviation from the normal physical state and avoid an adverse health event [11]. In this framework, disease risk can be viewed as a proxy indicator of the whole-body robustness [12]. Physical resilience could be defined as the ability to bounce back, and quickly and completely recover after an adverse health event [11, 12]. The ability to survive to very old age could be used as a proxy indicator of the whole-body resilience [11, 12]. Physical resilience universally declines with age, increasing vulnerability to death as people grow older. Robustness also generally declines with age – manifested by increased risks of many health disorders (e.g. Alzheimer’s disease, stroke, renal and heart failure, fractures, pneumonia), and disabilities in the elderly; however, robustness may improve in some health domains, reflected in declining risks of certain chronic diseases (e.g. several cancers, diabetes, asthma) towards extreme ages [11–13]. This indicates that the mechanisms underlying changes in physical robustness and resilience during aging are not necessarily (or entirely) the same. The same factor may have beneficial effects on resilience and adverse effects on robustness, and vice versa. For example, a chronically suppressed apoptosis in the body may contribute to both increased risk of cancer (lower robustness) in middle-old life and better survival at extreme ages (higher resilience) [14].

The long-lived individuals may live longer than the general population for various reasons. They may be more robust than the general population because they are able to avoid major diseases, or they may be more resilient because they are able to better survive after disease onset, or both. They may also live longer simply ‘by chance’. This study investigates whether long-lived female siblings are more robust and/or resilient than sporadic (“non-familial”) long-lived women of the same age, and, specifically, whether they have a better ability to (i) avoid the common diseases of the elderly at ages 65+ (proxy for robustness), and/or (ii) survive to extreme ages (proxy for resilience). We compared the cumulative incidence of the 20 most prevalent chronic conditions from age 65 between long-lived female siblings and sporadic long-lived Danish women. We also evaluated the difference in overall survival at the oldest-old ages between the familial and the sporadic long-livers, and examined factors contributing to this difference. Due to a scarce number of long-lived males, we used only data on long-lived females from three studies enrolling Danish long-lived siblings.

## RESULTS

### Study population

The study population was comprised of 2,312 long-lived women: 1,156 female siblings and 1,156 controls. The median age on January 1^st^, 2006 was 91.3 years (range: 68.8-105.0), with a median age of 91.7 for siblings and 90.9 for sporadic long-lived women (p=0.013). These women were mainly widowed (79.4% in siblings and 76.7% in controls (Table 1)). Nearly two thirds of our population had a *null* Charlson Comorbidity Index (CCI) score, and the numbers were 63.2% and 60.1% for siblings and controls (p=0.282), respectively. The prevalence of the 20 chronic conditions is shown in Table 1. The most prevalent condition was hypertensive disease (51.7% in siblings vs. 56.0% in controls, p=0.041) followed by respiratory allergy (42.8 vs. 43.4, p=0.769), cataract (39.1 vs. 39.5, p=0.831), hearing loss (38.4 vs. 33.4, p=0.013) and depression (32.1 vs. 38.9, p=0.001). If the Bonferroni correction or the false discovery rate (FDR) test [15] was used to account for multiple testing in Table 1, only the lower risk of depression among siblings remained statistically significant.

**Table 1:** Characteristics of study population.

	**Study population (n=2,312)**		**Siblings (n=1,156)**		**Controls (n=1,156)**		**p-value^a^**
	**n**	**(%)**	**n**	**(%)**	**n**	**(%)**	
**Marital status**							**<0.001**
Unmarried	161	(7.0)	91	(7.9)	70	(6.1)	
Married	198	(8.6)	105	(9.1)	93	(8.0)	
Divorced	148	(6.4)	42	(3.6)	106	(9.2)	
Widowed	1,805	(78.0)	918	(79.4)	887	(76.7)	
**Charlson's comorbidity index**							0.282
0	1,426	(61.7)	731	(63.2)	695	(60.1)	
1-2	759	(32.8)	362	(31.3)	397	(34.3)	
≥ 3	127	(5.5)	63	(5.5)	64	(5.6)	
**Specific comorbidities**							
Disturbance in lipoprotein circulation and other lipids	115	(5.0)	54	(4.7)	61	(5.3)	0.503
Diabetes	80	(3.5)	43	(3.7)	37	(3.2)	0.495
Choroid and retina disorders	109	(4.7)	61	(4.6)	56	(4.8)	0.768
Diseases of eye lens (cataracts)	909	(39.3)	452	(39.1)	457	(39.5)	0.831
Glaucoma	298	(12.9)	152	(13.2)	146	(12.6)	0.710
Hearing loss	831	(35.9)	444	(38.4)	387	(33.4)	**0.013**
Hypertensive diseases	1,245	(53.9)	598	(51.7)	647	(56.0)	**0.041**
Atrial fibrillation and flutter	163	(7.1)	71	(6.1)	92	(8.0)	*0.088*
Ischemic heart diseases	304	(13.2)	159	(13.8)	145	(12.5)	0.389
Cerebrovascular diseases	330	(14.3)	151	(13.1)	179	(15.5)	*0.096*
Respiratory allergy	997	(43.1)	495	(42.8)	502	(43.4)	0.769
Chronic low respiratory diseases	403	(17.4)	181	(15.7)	222	(19.2)	**0.025**
Chronic Obstructive Pulmonary Disease	80	(3.5)	33	(2.9)	47	(4.1)	0.111
Asthma	33	(1.4)	14	(1.2)	19	(1.6)	0.381
Ulcers	156	(6.8)	78	(6.8)	78	(6.8)	1.000
Osteoporosis	292	(12.6)	159	(13.8)	133	(11.5)	0.104
Arthrosis	338	(14.6)	168	(14.5)	170	(14.7)	0.906
Depression	821	(35.5)	371	(32.1)	450	(38.9)	0.001^*^
Dementia	127	(5.5)	54	(4.7)	73	(6.3)	*0.083*
Cancer	395	(17.1)	209	(18.1)	186	(16.1)	0.204
**Medication prescription**							0.396
Yes	1,702	(73.6)	842	(72.8)	860	(74.4)	
No	610	(26.4)	314	(27.2)	296	(25.6)	
**Treated organs**							
Alimentary tract and metabolism	668	(28.9)	325	(28.1)	343	(29.7)	0.409
Blood and blood forming organs	468	(20.2)	230	(19.9)	238	(20.6)	0.679
Cardiovacular system	882	(38.2)	430	(37.2)	452	(39.1)	0.346
Dermatologicals	98	(4.2)	43	(3.7)	55	(4.8)	0.215
Genito urinary system and sex hormones	106	(4.6)	49	(4.2)	57	(4.9)	0.426
Systemic hormonal preparations	143	(6.2)	69	(6.0)	74	(6.4)	0.666
Anti-infectives for systemic use	243	(10.5)	114	(9.9)	129	(11.2)	0.309
Antineoplasic and immunomodulating agents	9	(0.4)	3	(0.3)	6	(0.6)	0.316
Musculo-skeletal system	256	(11.1)	133	(11.5)	123	(10.6)	0.507
Nervous system	965	(41.7)	459	(39.7)	506	(43.8)	**0.047**
Antiparasidic, insecticides and repellents	45	(2.0)	18	(1.6)	27	(2.3)	0.175
Respiratory system	174	(7.5)	82	(7.1)	92	(8.0)	0.430
Sensory organs	241	(10.4)	111	(9.6)	130	(11.3)	0.196

^a^ A p-value followed by an asterix (*) indicates a statistically significant finding after adjustment for multiple testing using the Bonferroni correction or FDR-test.

The majority of the women reported medication prescription within the previous month - as median number of prescribed drugs (on average two; range: 0-16). The proportion of siblings and controls receiving prescriptions was similar (72.8% and 74.4%, respectively, p=0.396); however, on average, siblings received a lower number of distinct drugs than controls (p=0.047). The prescribed drugs were mainly related to the nervous systems (39.7% in siblings vs. 43.8% in controls, p=0.047) and cardiovascular (37.2% vs. 39.1%, p=0.346).

### Cumulative incidence

In the unadjusted models, long-lived siblings had a lower risk of hypertensive disease (OR=0.84, 95%CI=0.71-0.99), chronic low respiratory diseases (CLRD) (OR=0.79, 95%CI=0.64-0.97), and depression (OR=0.74, 95%CI=0.62-0.88) than sporadic long-livers (Table 2). They also presented a tendency towards lower cumulative incidence of atrial fibrillation and flutter (OR=0.75 95%CI=0.54-1.04), cerebrovascular diseases (OR=0.80 95%CI=0.64-1.03) and dementia (OR=0.73 95%CI=0.51-1.04). On the other hand, siblings had a higher cumulative incidence of hearing loss (OR=1.24 95%CI=1.05-1.47) compared to the controls. After adjustment for age, the long-lived female siblings still had a significantly lower risk of hypertensive disease (OR=0.82 95%CI=0.68-0.99) and depression (OR=0.72 95%CI=0.60-0.88) compared to the sporadic long-livers. They also had a significantly lower risk of cerebrovascular diseases (OR=0.73 95%CI=0.55-0.96). The risk of hearing loss did not remain significant after adjustment for age. However, the risk of cancer became higher (OR=1.27 95%CI=0.99-1.64) in long-lived female siblings compared to the sporadic long-livers after adjustment for age, though with marginal significance. If the Bonferroni correction or the FDR test was used to account for multiple testing, only the association with depression remained statistically significant.

**Table 2:** Risk of presenting chronic conditions from age 65 and over among long-lived female siblings compared to sporadic long-lived Danish women, results from conditional logistic models, n=2,352.

**Chronic conditions**	**Unadjusted models**			**Models adjusted for age**		
	**OR**	**95% CI**	**p-value^a^**		**OR**	**95% CI**	**p-value^a^**
Cancer	1.15	(0.93-1.43)	0.202		***1.27***	***(0.99-1.64)***	***0.059***
Hypertensive diseases	***0.84***	***(0.71-0.99)***	***0.036***		***0.82***	***(0.68-0.99)***	***0.035***
Atrial fibrillation and flutter	0.75	(0.54-1.04)	0.082		0.79	(0.55-1.14)	0.213
Ischemic heart diseases	1.11	(0.87-1.41)	0.860		1.03	(0.78-1.35)	0.862
Cerebrovascular diseases	0.81	(0.64-1.03)	0.088		***0.73***	***(0.55-0.96)***	***0.025***
Diabetes	1.20	(0.74-1.88)	0.480		1.46	(0.82-2.61)	0.199
Disturbance in lipoprotein circulation and other lipids	0.88	(0.60-1.28)	0.495		0.92	(0.61-1.40)	0.711
Depression	***0.74***	***(0.62-0.88)***	0.001^*^		***0.72***	***(0.60-0.88)***	0.001^*^
Dementia	0.73	(0.51-1.04)	0.085		0.73	(0.48-1.13)	0.162
Chronic low respiratory diseases	***0.79***	***(0.64-0.97)***	***0.028***		0.85	(0.67-1.07)	0.169
Chronic Obstructive Pulmonary Disease	0.70	(0.44-1.09)	0.115		0.68	(0.42-1.12)	0.128
Asthma	0.74	(0.37-1.45)	0.386		0.85	(0.39-1.85)	0.681
Respiratory allergy	0.98	(0.83-1.15)	0.773		1.11	(0.92-1.33)	0.269
Choroid and retina disorders	0.94	(0.65-1.38)	0.772		0.94	(0.60-1.45)	0.764
Diseases of eye lens (cataracts)	1.02	(0.86-1.20)	0.831		0.92	(0.76-1.11)	0.381
Glaucoma	1.05	(0.82-1.34)	0.709		1.08	(0.81-1.44)	0.598
Hearing loss	***1.24***	***(1.05-1.47)***	***0.013***		1.14	(0.94-1.39)	0.189
Ulcers	1.00	(0.72-1.38)	1.000		0.90	(0.62-1.31)	0.581
Osteoporosis	1.23	(0.96-1.58)	0.102		1.28	(0.96-1.69)	0.090
Arthrosis	0.99	(0.78-1.24)	0.907		0.84	(0.64-1.10)	0.201

^a^ A p-value followed by an asterix (*) indicates a statistically significant finding after adjustment for multiple testing using the Bonferroni correction or FDR-test.

### Survival

**Figure 1:**
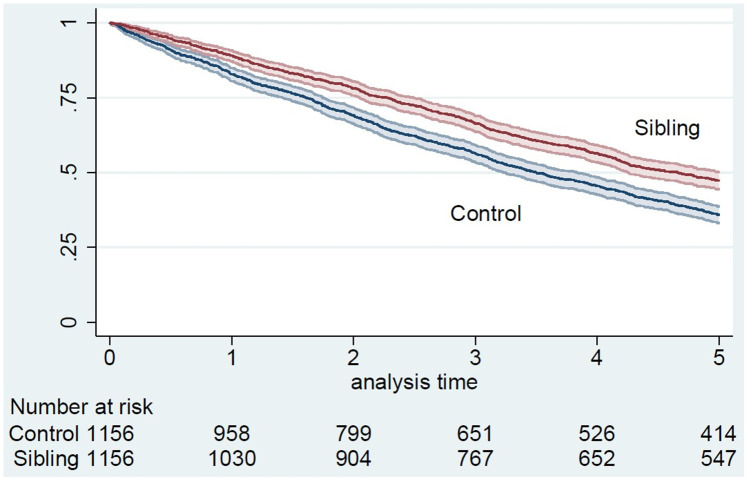
**5-year survival of siblings and controls from January 1^st^, 2006, Kaplan-Meier curve.**

At the end of the follow-up, 1,763 (76.3%) women, 833 (72.1%) siblings, and 930 (80.4%) controls were deceased. Overall survival for siblings and controls was 89% and 83% at 1 year, 66% and 56% at 3 years, and 47% and 36% at 5 years (p<0.001), respectively (Figure 1).

Long-lived female siblings had better 5-year overall survival than sporadic long-lived women (hazard ratio (HR)=0.71 95%CI=0.63-0.81) (Table 3). After adjustment for age, the HR of death among siblings compared to controls was lower (HR=0.68 95%CI=0.58-0.79), while it increased after adjustment for marital status (HR=0.72 95%CI=0.63-0.82), cerebrovascular diseases (HR=0.72 95%CI=0.63-0.82), depression (HR=0.73 95%CI=0.64-0.83), number of prescribed drugs (HR=0.72 95%CI=0.63-0.82) or nervous system drugs prescriptions (HR=0.72 95%CI=0.63-0.82), and it remained unchanged after adjustment for hypertensive diseases (HR=0.71 95%CI=0.63-0.81). In other words, the risk of death did not change or change only marginally after the adjustment, and remained significant for all covariates. Thus, after adjustment for all the variables simultaneously, female siblings still had significantly better total survival than controls (HR=0.72, 95%CI=0.62-0.85).

**Table 3:** Risk of death in high age among long-lived female siblings compared to sporadic long-lived Danish women, results from stratified Cox models, n=2,352.

**Models**	**HR**	**95% CI**	**p-value**
Without covariate	0.71	[0.63-0.81]	<0.001
Including age	0.68	[0.58-0.79]	<0.001
Including marital status	0.72	[0.63-0.82]	<0.001
Including hypertensive diseases	0.71	[0.63-0.81]	<0.001
Including cerebrovascular diseases	0.72	[0.63-0.82]	<0.001
Including depression	0.73	[0.64-0.83]	<0.001
Including no. of prescribed drugs	0.72	[0.63-0.82]	<0.001
Including nervous system drugs prescription	0.72	[0.63-0.82]	<0.001
Including age, marital status	0.69	[0.60-0.81]	<0.001
Including hypertensive diseases, cerebrovascular diseases, depression	0.73	[0.64-0.83]	<0.001
Including age, marital status, all the previous chronic conditions	0.72	[0.62-0.84]	<0.001
Including age, marital status, all the previous chronic conditions, no. of prescribed drugs	0.72	[0.62-0.85]	<0.001

## DISCUSSION

People may live long for various reasons including better robustness or better resilience, or both. They may also live longer lives simply by chance. In this study, we examined whether the long-lived female siblings are more robust and/or more resilient than sporadic long-lived women. Our results indicate that the long-lived siblings may be more robust to some health conditions (hypertension, cerebrovascular disease, depression), and less robust to some other (cancer); though only the risk of depression (OR=0.72; p-value=0.001) remained statistically significant after adjustment for multiple testing [15]. Our results strongly support the possibility that the long-lived female siblings are more resilient than sporadic long-livers because they show significantly better survival at extreme ages, even after controlling for all covariates/comorbidities. This indicates that familial longevity could be mainly related to a better resilience as the ability to overcome various life and health problems, rather than to a simply good health, and that being not depressed may be a key factor supporting organism’s robustness in advanced years of life.

Findings from earlier US studies that used samples of LLFS data suggest that long-lived siblings could be more robust to some diseases. Ash et al. [8] found lower risks of depression, Alzheimer’s disease, diabetes, heart failure, and some other conditions, in the long-lived LLFS siblings compared to the sporadic long-lived Medicare beneficiaries [8]. Unlike Ash et al., we found no evidence for a significantly different risk of dementia between the long-lived female siblings and controls [8]. However, Ash et al. studied a combination of both sexes, whereas our study focused only on females, which might lead to the difference in results. Similar to our findings, Newman et al. reported a lower risk of hypertension in LLFS compared to the Framingham Heart Study (FHS), but not to the Cardiovascular Health Study (CHS) and the New England Centenarian Study (NECS) participants [9]. That study also reported mixed results regarding stroke, with a higher risk in long-lived LLFS siblings compared to CHS and FHS, and no significant difference for NECS participants [9], while our results indicated a lower risk of cerebrovascular disease in the long-lived female siblings in the age-adjusted analysis.

In our study, we found a marginally significant higher risk of cancer among the long-lived female siblings compared to sporadic long-livers. Ukraintseva et al. earlier suggested potential biological mechanisms of trade-offs between extreme longevity and cancer risk, including antagonistic pleiotropic role of some genetic and non-genetic factors in aging and cancer development, which could be applicable to these results [14]. Other studies did not report a significant difference in the risk of cancer between long-lived LLFS siblings and controls [8, 9], but this may be due to differences in research design. A higher risk of hearing loss in the long-lived female siblings compared to sporadic long-livers did not remain significant after adjustment for age. So, we may assume that this difference was related to the age difference between siblings and controls. If multiple testing was considered using the conservative Bonferroni correction or the FDR test, only the lower risk of depression among siblings remained statistically significant [15].

Regarding survival to extreme ages, we observed a better overall survival of female long-lived siblings compared to sporadic long-livers. Similar findings were reported in a Dutch study that also showed better survival of the long-lived siblings at high ages, as compared to long-lived sporadic controls [10]. The association remained statistically significant after adjustment for age, marital status, comorbidities and/or medication.

It should be emphasized that none of the above studies considered indicators of physiological robustness and resilience in the same population, whereas in our large population-based study, we investigated both disease risks and survival to extreme ages in the same individuals. Our results suggest that better physiological robustness (manifested by lower disease risks) does not entirely explain better resilience (manifested by a higher survival to extreme ages) of female members of the long-lived families, as compared to sporadic long-livers. And adjustment for the above diseases, to which the long-lived female siblings were more robust, did not change the association, or did so only marginally.

We showed that longevity clustered in families can lead to a significantly lower mortality risk at extreme ages compared to sporadic long-livers. This suggests that resilience has similar biological mechanisms in the long-lived siblings influenced by common genetic and other familial factors. Indeed, in the first part of their lives, siblings share many environmental factors (e.g., socioeconomic status, place of residence, lifestyle). In addition, studies have highlighted the influence of genetic effects on lifespan at older ages, indicating that genetic factors could play a major role in the familial longevity [6]. Genetic factors are more likely than other factors to be related to resilience in long-lived siblings compared to sporadic long-livers, although other factors should also be explored.

There are some limitations to this study. First, long-lived female siblings were, on average, slightly but significantly older than the sporadic long-lived women even after matching on age. However, our analyses were adjusted for age. This adjustment did not lead to different results in the survival analyses. In fact, the adjustment for age only changed the HR slightly, and the association became stronger. Second, the condition or disease for which the medication was prescribed (indication code) was not available [16]. Not considering the indication codes could lead to a potential information bias with an overestimation of some chronic conditions. Nonetheless, if there was information bias, it was non-differential with an overestimation in long-lived siblings and controls. Third, only chronic conditions diagnosed through medication or in hospitals were considered. Consequently, some conditions could not be included. However, we focused on the 20 most prevalent chronic conditions in Danes aged 75 and over [17] and defined them based on validated definitions [16]. Finally, the initial study, which in 2004 identified the long-lived families that were included in the present study, did not use current definitions of longevity such as top sex and birth cohort survival percentiles [18, 19]. In our study population, 99.5% of the recruited families included at least two siblings who survived to age 90 years, whereas for the remaining families, one sibling survived well past 90 years and at least one other sibling survived to age 89 years. With our definition of longevity, a large sibship – everything else equal – has a higher probability of becoming a long-lived family compared to a sibship of say two. Recognizing this bias, we have been collecting evidence to address the potential size of the bias. We found that the long-lived siblings in our study were from sibships with an average of 7.2 siblings compared to 6.6 in 358 control families that were selected among families with at least two children. A difference this size seems unlikely to be of importance for late life disease and survival. Moreover, we cannot exclude that some controls were from non-identified long-lived families, but they would be rare. Also, males have higher mortality throughout life compared to females. Therefore, a study of similar long-lived men would have been an even more selected study sample and bring additional information to our findings. However, in our study we were not able to identify a sufficient number of male controls in the 5% sample of the Danish population to allow meaningful analyses.

The main strength of this work lies in the study design consisting of long-lived female siblings matched on age with female controls from a representative sample of the Danish population. Thus, the registry-based study design permitted avoidance of selection bias. Long-lived siblings and controls were all identified in high-quality National Danish registers, also leading to a large sample size. Finally, the definition of chronic conditions was based on both hospital and medication data [16]. In this way, we identified individuals with different disease levels, although not all individuals.

In conclusion, long-lived female siblings demonstrated better robustness to some health conditions (especially, depression), while increased vulnerability to some other diseases (cancer). Physiological resilience (manifested in higher chances of survival to extreme ages) was consistently better in the long-lived female siblings than in their age-peers from general population. This indicates that the ability to overcome deleterious life events may be more important for extreme longevity than a good health alone, and that avoiding depression is major factor of maintaining physical robustness in familial longevity. A consistently better survival of the long-lived siblings also suggests that resilience may have stronger genetic component in familial longevity, warranting further investigation. Overall, results of this study indicate that long-lived siblings are excellent candidates for healthy longevity studies, and that familial longevity cases could be more informative than sporadic cases for studying mechanisms of longevity. Since health phenotypes such as discharge diagnoses of chronic conditions and medication could not explain better resilience of the long-lived female siblings, a next step can be to focus on other factors that might explain their better resilience, such as response to acute health events, dynamic changes in functional status, cognitive functioning, or psychological factors [11; 20–22]. Cognitive and physical functioning are known to be highly predictive of survival in the very old individuals [20]. In addition, it would be reasonable to explore last year of life events, or the cause of death in long-lived siblings compared to sporadic long-livers to examine whether they experience different life events compared to sporadic long-lived women.

## MATERIALS AND METHODS

### Study population

The identification of long-lived siblings was undertaken in three nationwide, consecutive studies in Denmark, for which recruitment ran sequentially during the years 2004 to 2009: the Danish Oldest Siblings (DOS) pilot study, the Genetics of Healthy Ageing (GeHA) study [23], and the Danish part of the Long Life Family Study (LLFS) [24]. All individuals born before April 2, 1918, and alive in 2004 were identified in the Danish Civil Registration System (CRS). Long-lived siblings were defined in different ways depending on the study. Recruitment to DOS was conditional on both siblings being alive and 88 years or older; recruitment to GeHA required both siblings to be alive and above age 90, and the LLFS recruited only families with a family longevity index (FLoSS) score above 7 [24]. In all, 3,972 siblings from 659 families were enrolled in either DOS, GeHA, or LLFS, with 659 siblings from 114 families in DOS, 2,736 siblings from 469 families in GeHA, and 577 siblings from 76 families in LLFS.

Long-lived female siblings enrolled in these studies and alive on January 1^st^, 2006 were included. Each female sibling was matched with one female control from the Danish population alive on January 1^st^, 2006 and randomly selected on age (+/- 2 years).

### Danish national population-based registers

The information used in this study was mainly extracted from the Danish national population-based registers presented below.

### The Danish civil registration system (CRS)

The CRS, which covers the entire population alive and residing in Denmark since April 2, 1968, contains information on each resident’s vital status, sex, place and date of birth as well as familial links (e.g. parents, siblings, spouse) [25, 26]. All persons registered in CRS are assigned a unique personal identification number which is used in all national registers, enabling accurate linkage between all national registers. Once a person has been assigned a unique personal identification number, the same number will not be assigned to other persons and this number follows the person afterwards.

### Health registers

The Danish National Patient Register (NPR) is a health register established in 1977 [27, 28]. The NPR covers inpatients and somatic wards as well as outpatients and psychiatric wards since 1995. The reported data are administrative (e.g. patient’s municipality, identification of hospital ward, date and time of activity, and information on accidents leading to hospital contact) and clinical (e.g. diagnoses and surgical procedures). Different types of diagnoses are recorded: primary diagnoses (main reason for hospitalization), secondary diagnoses (supplementing the primary diagnosis), referral diagnoses (reason for referral), temporary diagnoses and complications.

The Danish Cancer Registry (DCR) contains records of all incidences of malignant neoplasms in the Danish population from 1943 onwards [29]. The register is considered almost complete and has a high degree of validity [29, 30].

The Danish National Prescription Registry (DNPR) provides individual-level information on dispensed prescriptions for each person resident in Denmark since 1995 [31, 32]. DNPR contains information on all prescription drugs dispensed at Danish community pharmacies as well as prescriptions dispensed to residents of long-term care institutions (e.g. nursing homes). The register records information related to drug user and to prescriber, as well as drug and pharmacy information.

### Outcomes

The first outcome was the cumulative incidence from age 65 of the 20 most prevalent chronic conditions in Danes aged 75 and older [17]: *cancers, hypertensive disease, atrial fibrillation and flutter, ischemic heart disease (including myocardial infarction), cerebrovascular diseases (including stroke), diabetes, disturbance in lipoprotein circulation and other lipids, depression, dementia, chronic low respiratory diseases (CLRD), chronic obstructive respiratory disease (COPD), asthma, respiratory allergy, choroid and retina disorders, diseases of eye lens (cataract), glaucoma, hearing loss, ulcers, osteoporosis, arthrosis*. Except for cancers, all diseases were identified through the NPR and/or the DNPR using the register-based definitions defined by Hvidberg et al [16] (Supplementary Table 1). Cancers were identified using the DCR.

The second outcome was overall survival. Survival time was calculated from January 1^st^, 2006 to the date of death, emigration or to the date of last follow-up (July 1^st^, 2013), whichever came first. All-cause mortality was defined as death from any cause. Patients still alive were censored at the date of last follow-up.

### Covariates

Analyses were adjusted for the following factors, usually associated with overall survival in older adults: marital status, Charlson’s comorbidity index (CCI [33]), medication prescription and organs treated by prescription drugs. Marital status was considered on January 1^st^, 2006 from the CRS. The CCI was constructed from hospital data in the 10 years prior to January 1^st^, 2006. It was based on primary and secondary disease diagnoses recorded in the NPR. In order to capture medical habits and general health in addition to CCI, medication prescription (yes versus no), the number of prescribed drugs and treated organs within the month prior to January 1^st^, 2006 were considered. The treated organs referred to level 1 Atomic Therapeutic Chemical groups concerned by medication prescription. Prescribed drugs were assessed to evaluate polypharmacy and as a proxy for general health in addition to CCI.

### Statistical analyses

The cumulative incidence between female siblings and controls was compared by performing conditional logistic regression models based on matching data.

Survival time from January 1^st^, 2006 was described with Kaplan-Meier curves for female siblings and controls separately and compared using the logrank test. Survival analyses were performed using stratified Cox proportional hazards models based on the matching data with and without potential confounding covariates. Proportional-hazards assumption was tested using Schoenfeld residuals.

The study has been approved by The Regional Scientific Ethical Committees for Southern Denmark (S-VF-20030227) and The Danish Data Protection Agency (# J.nr. 2008-41-1753).

## Supplementary Material

Supplementary Table 1
