# Supplementary material for: Physical robustness and resilience among long-lived female siblings: a comparison with sporadic long-livers
Source: Aging (Albany NY). 2020 Jul 11;12(14):15157–68. doi: 10.18632/aging.103618 (PMC7425496; doi:10.18632/aging.103618)
Supplement: Supplementary Table 1 [file aging-12-103618-s001..pdf]

SUPPLEMENTARY TABLE

Supplementary Table 1. Coding of the 20 most prevalent conditions in Danes aged 75+ on National Danish databases.

No.	Conditions	Databases*	Definition
1	**Malignant neoplasms**	DNC	All except ICD-10 C44
	**Endocrine nutritional and metabolic diseases**		
2	Disorders of lipoprotein metabolism / other lipidemias (e.g. hypercholesterolaemia, hyperglyceridaemia, hyperlipidaemia)	NPR	ICD-8: 279<br>ICD10: E78
		DNPR	ATC: C10
3	Diabetes<br>Diabetes type 1<br>Diabetes type 2<br>Diabetes others	NPR	ICD-8: 249, 250<br>ICD-10: E10-E14
		DNPR	ATC: A10A, A10B.<br>At least two prescriptions.
	**Diseases of the eye and adnexa and diseases of the ear and mastoid process**		
4	Choroid and retina disorders	NPR	ICD-8: 367, 376, 377.0-377.4<br>ICD-10: H31, H32, H34, H35
5	Diseases of eye lens (cataracts)	NPR	ICD-8: 374<br>ICD-10: H25-H28
6	Glaucoma	NPR	ICD-8: 375<br>ICD-10: H40-H42
		DNPR	ATC: S01E
7	Hearing loss	NPR	ICD-8: 389, 781.3<br>ICD-10: H90, H910, H912, H913, H918, H930, H932, H933, H911, H919
	**Diseases of the circulatory system**		
8	Hypertensive diseases	NPR	ICD-8: 400-404<br>ICD-10: I10-I15
		DNPR	Combination treatment with at least two of the following classes of hypertensive drugs with ATC codes:<br>- α Adrenergic blockers (C02A, C02B, C02C)<br>- Non-loop diuretics (C02DA, C02L, C03A, C03B, C03D, C03E, C03X, C07C, C07D, C08G, C09BA, C09DA, C09XA52)<br>- Vasodilators (C02DB, C02DD, C02DG, C04, C05)<br>- β Blockers (C07)<br>- Calcium channel blockers (C07F, C08, C09BB, C09DB)<br>- Renin-angiotensin system inhibitors (C09)
9	Atrial fibrillation and flutter	NPR	ICD-8: 427.93, 427.94<br>ICD-10: I48
10	Ischaemic heart diseases (including myocardial infarction)	NPR	ICD-8: 410-414<br>ICD-10: I20-I25
11	Cerebrovascular diseases (including stroke)	NPR	ICD-8: 430-438<br>ICD-10: I60-69, G45, G46<br>DZ501 A-diagnosis in combination with I61, I63-64 as A- or B- diagnosis
	**Diseases of respiratory system**		
12	Respiratory allergy	NPR	ICD-8: 507.00-507.03, 507.08, 507.09<br>ICD-10: J30 except J30.0
		DNPR	ATC: V01AA02, V01AA03, V01AA05, V01AA11, R01AC, R01AD, R06A, S01G, R01BA52
13	Chronic lower respiratory diseases	NPR	ICD-8: 490-492<br>ICD-10: J40-43, J47

		DNPR	ATC: R03AC, R03AK, R03BA, R03BB, R03CC, R03DA, R03DC, V03AN01<br>Except if cystic fibrosis (ICD-8 273.0, ICD-10 E84) or COPD medication or asthma specific medication
14	COPD	NPR	ICD-8: 491, 492<br>ICD-10: J44
		DNPR	ATC: R03AC18, R03AC19, R03AL02, R03AL03, R03AL04, R03BB04, R03BB05, R03BB06, R03DX07<br>Except if cystic fibrosis (ICD-8 273.0, ICD-10 E84)
15	Asthma	NPR	ICD-8: 493<br>ICD-10: J45-J46
		DNPR	ATC: R03DC03<br>Except if cystic fibrosis (ICD-8 273.0, ICD-10 E84)
	**Diseases of digestive system**		
16	Ulcers	NPR	ICD-8: 531-534<br>ICD-10: K25-K27
		DNPR	ATC: A02BD
	**Diseases of the musculoskeletal system and connective tissue**		
17	Osteoporosis	NPR	ICD-8: 723.0<br>ICD-10: M80-M81
		DNPR	ATC: M05BA01, M05BA04, M05BA06, M05BA07, M05BB01, M05BB03, G03XC01, H05AA02, H05AA03
18	Arthrosis	NPR	ICD-10: M15-M19
	**Mental and behavioural disorders**		
19	Depression	NPR	ICD-8: 296.09, 296.29, 296.99, 298.09, 300.49, 300.19<br>ICD-10: F32, F33, F34.1, F06.32
		DNPR	ATC: N06A
20	Dementia	NPR	ICD-8: 290.09, 290.10, 290.11, 290.12, 290.13, 290.14, 290.15, 290.16, 290.17, 290.18, 290.19, 293.09<br>ICD-10: F00, G30, F01, F02.0, F03.9, G31.8B, G31.8E, G31.9, G31.0B
		DNPR	ATC: N06D

* DNC: Danish Cancer Registry, NPR: Danish National Patient Register, DNPR: Danish National Prescription Register.
